# Comparative study of the load-bearing and deformation behaviors of metal mesh and polymer thin spray-on liner surface supports in mining roadways

**DOI:** 10.1371/journal.pone.0349272

**Published:** 2026-11-04

**Authors:** Han Liang, Kangdong Shi, Yunjing Shi, Zihan Bai, Daisong Liu, Chen Cao, Zedi Zhang

**Affiliations:** College of Mining, Liaoning Technical University, Fuxin, China; China University of Mining and Technology, CHINA

## Abstract

To investigate the differences in load-bearing capacity and deformation control between metal mesh and polymer thin spray-on liners (TSL) for roadway surface support, a study was conducted based on a mining roadway in Yulin, Shaanxi Province, China. Small-scale simulated roadway tests and engineering-scale numerical simulations were combined. Under the same geometric, boundary, and loading conditions, the peak load, strain response, surface displacement, and failure characteristics of the two support systems were compared. The effects of different surface supports on surrounding rock deformation and stress distribution were further analyzed under the same bolt–cable support conditions. The results show that the peak load of the metal mesh specimens was about 70 kN, with a maximum surface displacement of about 0.42 mm. The TSL specimens reached a peak load of about 85 kN, which was 21.4% higher than that of the metal mesh specimens, while the maximum surface displacement decreased by 23.8% to about 0.32 mm. Metal mesh mainly restrains loose rock blocks through tensile resistance, bending, and containment, whereas TSL provides earlier confinement to the shallow surrounding rock through continuous coverage, bonding, and membrane action. Numerical results indicate that, compared with metal mesh support, TSL support reduced the peak sidewall convergence, roof subsidence, and floor heave by 34.1%, 11.0%, and 6.3%, respectively, and slightly reduced the high-stress zone. The results suggest that, under the conditions investigated in this study, TSL has potential advantages in improving surface support performance and reducing shallow surrounding rock deformation. However, its long-term applicability and material safety require further verification through field monitoring and additional tests.

## Introduction

Spray-on surface support technology (TSL) is a novel surface support method that applies polymer-based or cementitious materials onto the surface of surrounding rock. It has the advantages of rapid construction, good continuity, high automation potential, and strong adaptability [1]. Compared with traditional metal mesh support, which requires processes such as mesh installation, overlapping, and fixing, TSL involves a simpler construction procedure, reducing mesh installation work and labor intensity. This feature meets the development requirements of rapid roadway excavation and integrated excavation-support systems in coal mines [2]. In terms of support timing, TSL exhibits relatively active confinement characteristics. After application, the spray layer can bond with the surrounding rock surface and penetrate into cracks, helping to bind loose rock masses. Therefore, it can participate in surface confinement at an early stage of deformation, which may limit crack propagation, reduce rock block loosening, and improve the integrity of the shallow surrounding rock [3]. During continuous deformation of the surrounding rock, TSL can interact with the shallow rock mass and contribute to a composite load-bearing response, providing confinement to the shallow rock and showing thin-shell or load-carrying membrane behavior under larger deformation conditions [4]. In contrast, metal mesh mainly restrains loosened or potentially detached rock blocks through tensile resistance, bending deformation, and containment effects [5]. Therefore, the main difference between the two surface support methods lies in their support mechanisms: TSL relies on continuous bonding and confinement, whereas metal mesh mainly provides discrete mechanical containment. TSL may have potential advantages in terms of early support participation and continuous surface confinement; however, its engineering performance is still affected by surrounding rock conditions, material properties, and the overall support system.

Early applications of TSL were mainly concentrated in hard-rock roadways in metal mines, where its primary function was to rapidly control the loosening and detachment of small rock blocks. Publicly reported engineering cases in which TSL replaces metal mesh in underground coal mines are still limited. One reason is that coal mine roadways generally experience greater deformation than hard-rock roadways, especially during mining-induced disturbance. Therefore, surface support materials need to provide adequate strength and bonding capacity while also accommodating deformation. In recent years, improvements in material performance have created favorable conditions for investigating the application of TSL in coal mine roadways. It should be noted that this study focuses on mechanical response and the evaluation of potential engineering application. No economic conclusions are drawn regarding material costs, labor costs, construction time, or life-cycle costs of TSL and metal mesh. A separate economic evaluation should be conducted based on field quantities and complete cost data.

The engineering performance of TSL in soft-rock and large-deformation roadways remains difficult to predict. First, the limited number of engineering applications provides insufficient practical experience for reference. Second, existing studies on TSL remain mainly at the stage of laboratory testing and numerical simulation. For example, Shan Z. et al. compared the support performance of TSL and metal mesh using cement plate tests [6]. Qiao Q. et al. investigated the support effect of TSL on hard-rock coal pillars using standard rock mechanical specimens, while Liang Han et al. analyzed the support performance of TSL and metal mesh for underground coal mine coal pillars through experimental simulations [7]. Kolapo et al. reported that TSL has high bonding strength and rapid curing characteristics, which may contribute to improved surrounding rock stability and construction efficiency [8]. Wang Shiming investigated the influence of TSL encapsulation support on sandstone spalling behavior and found that TSL can modify crack initiation and propagation through surface coating and interface bonding effects, thereby improving shallow rock stability. The study also indicated that TSL performance is closely related to spray thickness, encapsulation length, and impact loading conditions [9]. Kim systematically reviewed TSL materials, curing characteristics, mechanical properties, testing methods, and engineering applications, and pointed out that TSL exhibits relatively high tensile strength, bonding strength, and flexural capacity, making it a potential supplement to conventional surface support systems [10]. These studies have provided a foundation for the engineering application of TSL; however, reliable prediction of its field performance remains challenging.

Numerical simulation is a commonly used method for analyzing and predicting engineering performance. However, because publicly reported engineering applications of TSL in underground coal mines are still limited, and its material thickness, interface bonding characteristics, and deformation behavior differ significantly from those of conventional shotcrete, a unified and mature numerical representation method has not yet been established. Qiao Q. et al. [11] used liner structural elements to simulate the support effect of TSL. Liang Han et al. investigated the surface support mechanism of polymer thin spray-on liners through experimental and numerical studies, indicating that TSL and the constrained rock mass may form a thin-shell structure during large deformation, thereby contributing to cooperative load-bearing and deformation control of shallow surrounding rock [12]. Most existing structural elements are derived from numerical representations of shotcrete or lining structures. However, polymer thin spray-on liners typically have a thickness of only 5–8 mm, which differs considerably from concrete shotcrete layers thicker than 100 mm in terms of scale and deformation characteristics [13]. In addition, polymer thin spray-on materials generally exhibit good interface bonding properties and can penetrate joints and cracks in the surrounding rock, potentially forming a cooperative load-bearing structure and improving shallow rock stability [14]. Therefore, accurately representing the effects of thin-layer continuous coverage, interface interaction, and deformation compatibility in numerical models, and validating the major response characteristics through physical tests, remain key issues in TSL numerical research.

This study focuses on the question of how metal mesh surface support and continuous thin spray-on liner (TSL) support differ in terms of load-bearing behavior, deformation control, and confinement of shallow surrounding rock. First, under the same geometric, boundary, and loading conditions, simulated roadway tests with metal mesh and TSL surface support were conducted. The performance of the two support methods was compared based on peak load, surface displacement, strain evolution, and failure characteristics. Subsequently, an engineering-scale numerical model was established to compare the surrounding rock displacement and stress responses of metal mesh and TSL support under identical bolt–cable support conditions. This study mainly evaluates the relative differences caused by different surface support methods. It does not address material formulation optimization or propose a universal design equation for direct engineering application.

## Engineering background

This study was conducted based on a fully mechanized mining face of Hongliulin Mining Company in the Yulin mining area, northern Shaanxi Province, China. The mining face is located in the southern part of Panel 2 and mainly extracts the No. 4−2 coal seam. The average dip angle of the coal seam is 2°, representing a typical near-horizontal thick coal seam mining condition. As an active mining face, the panel is adjacent to a goaf on the mining side, while the auxiliary haulage roadway and solid coal pillar are located on the north and south sides, respectively. Therefore, the roadway is subjected to both relatively stable in-situ stress conditions and overlapping mining-induced disturbance effects.

As shown in Table 1, the coal seam at the working face is relatively stable, with an average thickness of 3.25 m. Based on the excavation data of the belt transportation roadway and the geological information from boreholes 9-HB10, L61, 8−1, and S5, the coal seam thickness ranges from 2.66 to 3.50 m, with a typical thickness of 3.35 m. Generally, one gangue layer is present within the coal seam, with a thickness of 0.05–0.10 m and a lithology of mudstone. The coal seam is classified as a stable seam.

**Table 1:** Coal seam conditions.

Coal Seam Thickness / m	Dip Angle / °	Average Thickness / m	Protodyakonov Coefficient	Mined Seam	Coal Type	Stability
2.6–3.5	0–$3^{\circ }$	3.25	1.0–2.5	4−2	Non-caking coal	Stable

As shown in Table 2, the roof and floor strata of the coal seam are relatively competent. The immediate roof consists of medium-grained sandstone with an average thickness of 2.18 m, while the main roof is composed of medium- to fine-grained sandstone with an average thickness of 6.35 m. The surrounding rock exhibits good integrity and relatively high strength. The floor consists mainly of sandy mudstone and fine-grained sandstone, showing relatively good stability.

**Table 2:** Lithology of coal seam roof and floor.

Roof/Floor	Rock Name	Thickness (m)	Rock Characteristics
Main Roof	Medium-fine sandstone	(4.69-8.02)/6.35	Gray, massive, medium-thick bedded, moderate sorting, good roundness, mainly quartz and feldspar, sub-angular, argillaceous-calcareous cementation, contains dark minerals, wavy bedding.
Immediate Roof	Medium-grained sandstone	(0.75-3.61)/2.18	Gray, wavy bedding, contains dark minerals, sub-angular, argillaceous-calcareous cementation, short columnar core. Locally fine sandstone roof.
Coal Seam	4−2 Coal	(2.6-3.5)/3.35	Black, massive, predominantly semi-dull to semi-bright, dull and vitrain bands, sparse endogenous fractures filled with calcite.
Immediate Floor	Sandy mudstone (Siltstone)	(1.6-1.88)/1.69	Gray, dark gray, massive, contains plant fossil fragments, short columnar core, RQD 51%.
Floor	Fine sandstone	(1.7-3.18)/2.44	Gray, dark gray fine sandstone, wavy bedding, contains dark minerals, sub-angular, argillaceous-calcareous cementation.

Under these geological conditions, the mining roadways were designed with a rectangular cross-section. The net width of the belt transportation and return air roadways is approximately 5.0 m, and the net height is approximately 3.4–3.5 m. In the field, a combined support system was adopted, consisting mainly of rock bolts and cable bolts, together with steel strips and metal mesh for surface control. The objective of this study is not to optimize the existing bolt–cable support parameters, but rather to compare the effects of metal mesh and TSL surface support on surrounding rock responses under the same basic bolt–cable support conditions. Therefore, the detailed support parameters used in the numerical analysis are presented collectively in Section 3.1.

Field monitoring and calculations based on the “broken zone theory” indicate that the development radius of the surrounding rock plastic zone is approximately 4.45 m, suggesting relatively stable surrounding rock conditions. According to mine pressure monitoring records, roadway deformation under similar geological conditions is effectively controlled. The roof separation warning and critical values are set at 20 mm and 40 mm, respectively, and the sidewall convergence remains within an acceptable range, meeting the safety requirements for fully mechanized mining operations.

In recent years, increasingly tight coordination between roadway development and mining operations has created greater demand for rapid excavation and reduced-manpower construction at the mine. Against this background, the mine plans to conduct field trials in which TSL partially replaces conventional metal mesh surface support, with the aim of evaluating the feasibility of the spray-on support process and its mechanical applicability, while providing technical preparation for future automated and reduced-manpower construction. It should be emphasized that this study evaluates only the mechanical response and construction feasibility of TSL. The available data are not sufficient to demonstrate that TSL can fully replace metal mesh under engineering conditions.

### Comparative experimental study of metal mesh and thin spray-on surface support in simulated roadways

The stability of roadway surrounding rock is influenced by multiple factors, including in-situ stress, rock mass properties, structural discontinuities, and support conditions. Scaled physical models can reproduce some of the key mechanical responses of underground structures under loading within controlled conditions. Therefore, they provide an effective approach for investigating surrounding rock deformation and failure mechanisms, analyzing support performance, and validating the major response characteristics obtained from numerical simulations.

The objective of the small-scale simulated roadway tests in this study was not to fully reproduce field conditions, but rather to highlight the primary mechanical behavior of the roadway surrounding rock–surface support system through reasonable simplification. The tests were used to compare the differences in shallow surrounding rock deformation evolution, local failure characteristics, and overall load-bearing capacity under different surface support conditions.

### Simulation test design

Physical model tests should reflect the key controlling factors of the investigated system in terms of geometric relationships, main load-transfer paths, and boundary constraints, so that the relative responses of different surface support methods can be compared. Based on the field roadway geometry and support conditions, two groups of simulated roadway tests were designed, including metal mesh surface support and TSL surface support, as shown in Fig 1.

**Fig 1:**
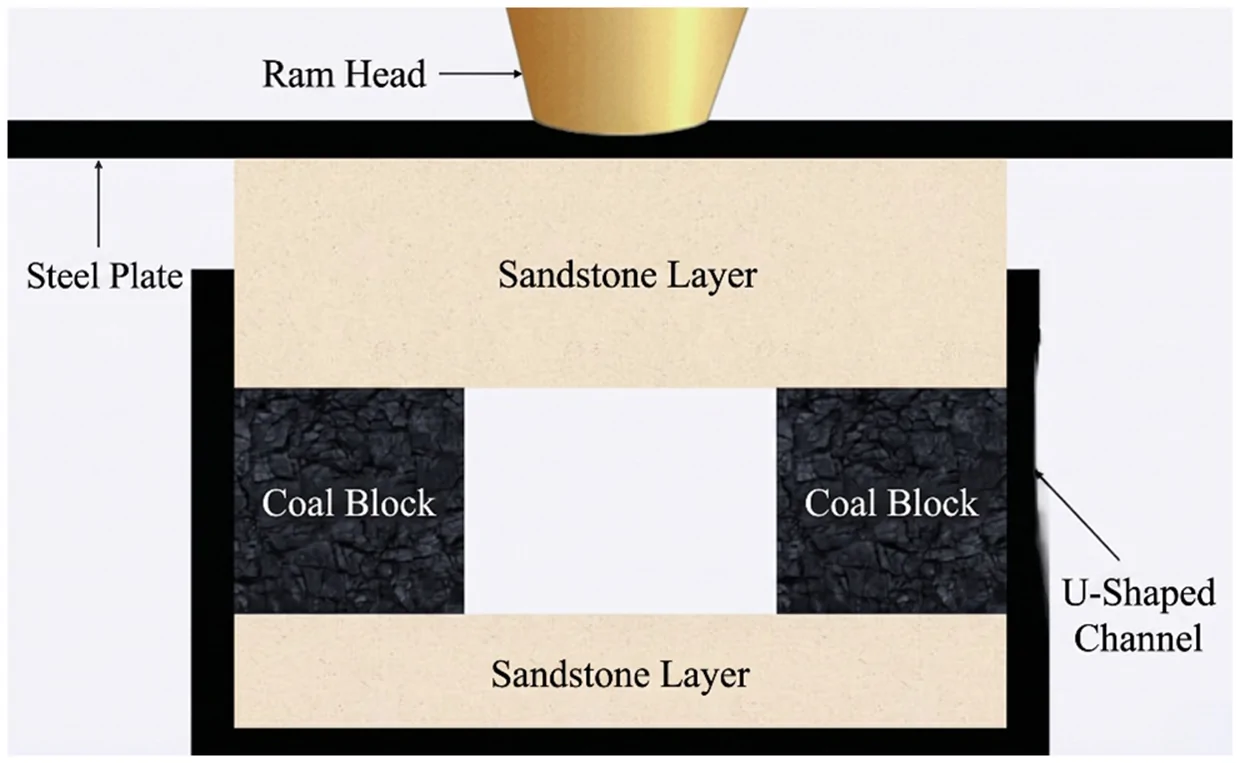
Schematic of the simulated roadway test.

The overall dimensions of the model were 205 mm (length) × 205 mm (width) × 150 mm (height). The roof consisted of a 60 mm-thick sandstone layer, and the floor consisted of a 30 mm-thick sandstone layer. The coal pillars on both sides had dimensions of 60 mm (height) × 60 mm (width) × 205 mm (length), forming an 85 mm-wide simulated roadway between the pillars. Based on the field roadway dimensions of 5.0 m × 3.4–3.5 m, the geometric scaling ratios corresponding to the roadway width and height were approximately 1:58.8 and 1:56.7–1:58.3, respectively. Therefore, a geometric similarity ratio of approximately 1:58 was adopted in this study.

Field measurements of the broken zone indicate that the disturbed depth in the shallow sidewall region is approximately 1.4–1.6 m. The lateral boundaries of the model were located about 60 mm from the roadway profile, corresponding to approximately 3.5 m at the field scale based on the above geometric similarity ratio. Therefore, the model boundaries were placed outside the main shallow disturbance zone. A U-shaped steel frame was used to provide rigid lateral constraints, and a steel loading plate larger than the roof specimen was applied to impose vertical loading, forming a repeatable lateral confinement–vertical loading path. This loading method was intended only to represent the main mechanical characteristics under increased advance abutment pressure during mining. It does not fully reproduce the in-situ horizontal stress, spatial evolution of mining-induced stress redistribution, or the complete progressive failure process observed in the field.

It should be noted that this test is a “geometrically similar mechanism comparison test” rather than a strictly complete similarity physical simulation. The model only maintains geometric similarity with respect to the roadway cross-section and major boundary distances. The strength, elastic modulus, TSL thickness, and mechanical parameters of the coal and rock materials were obtained from measured test values or predefined values. They were not simultaneously scaled according to unified similarity constants for strength, stiffness, density, and time scale. Time-dependent deformation characteristics, such as coal and rock creep, were also not considered. The loading condition consisted of rigid lateral confinement and monotonic vertical loading, which was used to represent the main mechanical characteristics associated with increased advance abutment pressure during mining. However, it cannot simultaneously reproduce the in-situ horizontal tectonic stress, spatial evolution of mining-induced abutment pressure, or the complete progressive failure process. Therefore, the model load, displacement, and failure time were not converted into absolute field-scale values. The tests were only used to compare the relative differences in load-bearing behavior, deformation, and failure characteristics of different surface support methods under identical boundary and loading conditions. The engineering-scale responses were further evaluated using an independent three-dimensional numerical model, as described in Section 4 (Table 3).

**Table 3:** Scaling relationships and applicability boundaries of the physical model.

Similarity parameter/Condition	Treatment adopted in this study	Applicability limitation
Geometric similarity ratio	The roadway cross-section was scaled at approximately 1:58, and the model boundary was located about 60 mm from the roadway profile.	Used to maintain the main spatial relationships and prevent the shallow disturbed zone from being directly affected by boundary constraints.
Strength similarity ratio	No unified strength similarity ratio was adopted. The coal and rock parameters were obtained from measured values of the test materials.	Not used to convert the model peak load into field-scale support capacity.
Stiffness similarity ratio	The elastic modulus of the coal and rock materials and the stiffness of the TSL were not scaled according to the geometric similarity ratio.	Used only to compare the relative differences between different surface support methods under identical specimen conditions.
Time similarity ratio	No time similarity relationship was established, and creep and long-term aging effects were not considered.	Not used to interpret failure time or extrapolate long-term service behavior.
Stress/loading path	Lateral confinement was provided by a U-shaped steel frame, and vertical loading was applied on the roof.	Provides an approximate representation of increased vertical abutment pressure but cannot reproduce the complete three-dimensional mining-induced stress path.

## Materials and methods

Sandstone was used for the roof and floor strata, while coal material was used for the sidewall pillars to construct the simulated roadway surrounding rock structure. To ensure the reliability of the material parameters, basic mechanical tests were conducted on the coal and rock materials before the experiments, including measurements of uniaxial compressive strength and elastic modulus. Based on the test results, the uniaxial compressive strength and elastic modulus of the roof and floor sandstone were set as 50 MPa and 30 GPa, respectively. For the coal pillars on both sides, the corresponding values were 5 MPa and 0.5 GPa, respectively. All specimens were prepared using the same coal and rock materials and identical structural configurations to ensure consistent surrounding rock conditions under different surface support conditions.

A U-shaped steel frame with a thickness of 10 mm was used to provide lateral confinement in the model tests. To reduce the influence of contact effects between the specimen and the steel frame, the inner surface of the frame was pre-coated with cement slurry, and sufficient contact between the model and the boundary was ensured during specimen assembly. Two groups of specimens were prepared, including a metal mesh surface support group and a polymer thin spray-on liner (TSL) surface support group. Except for the surface support type, all other conditions, including geometric dimensions, coal and rock material parameters, boundary constraints, and loading methods, were kept identical between the two groups (Table 4).

**Table 4:** Mechanical properties of materials used in the model tests.

Material	Uniaxial compressive strength (MPa)	Elastic modulus (GPa)	Source
Sandstone	50	30	Laboratory uniaxial compression tests
Coal blocks	5	0.5	Laboratory uniaxial compression tests
TSL	—	0.5	Material performance tests

The metal mesh group used diamond-shaped metal mesh to represent the conventional discrete surface support structure, which mainly provides containment and restraint for broken coal and rock blocks. The TSL group was prepared by uniformly spraying polymer slurry onto the roadway surface to form a continuous thin layer with a thickness of approximately 5 mm, representing the thin spray-on surface support used in engineering practice. The material parameters of the TSL were determined based on material performance tests and existing engineering application parameters, with consideration given mainly to its elastic deformation capacity, tensile properties, and interfacial bonding behavior (Fig 2).

**Fig 2:**
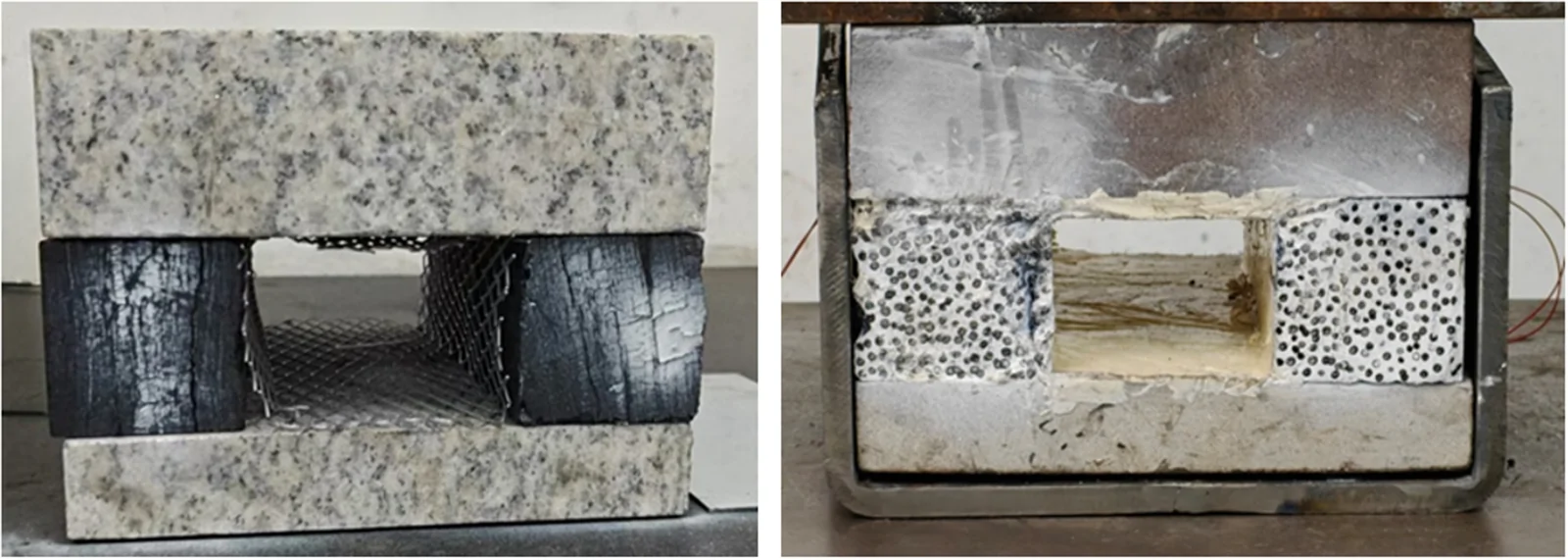
Metal mesh support and TSL support.

To evaluate the effects of different surface support methods on the load-bearing and deformation characteristics of roadway surrounding rock, a WAW-600C electro-hydraulic servo universal testing machine was used for loading. During the tests, digital image correlation (DIC) technology and resistance strain gauges were employed for full-process monitoring. The DIC technique was used to obtain full-field surface displacement and deformation evolution of the specimens, while the strain gauges were used to record local strain responses at key locations. These measurements were used to analyze the load-bearing characteristics and failure behavior of the coal–rock and surface support composite system under different surface support conditions (Fig 3).

**Fig 3:**
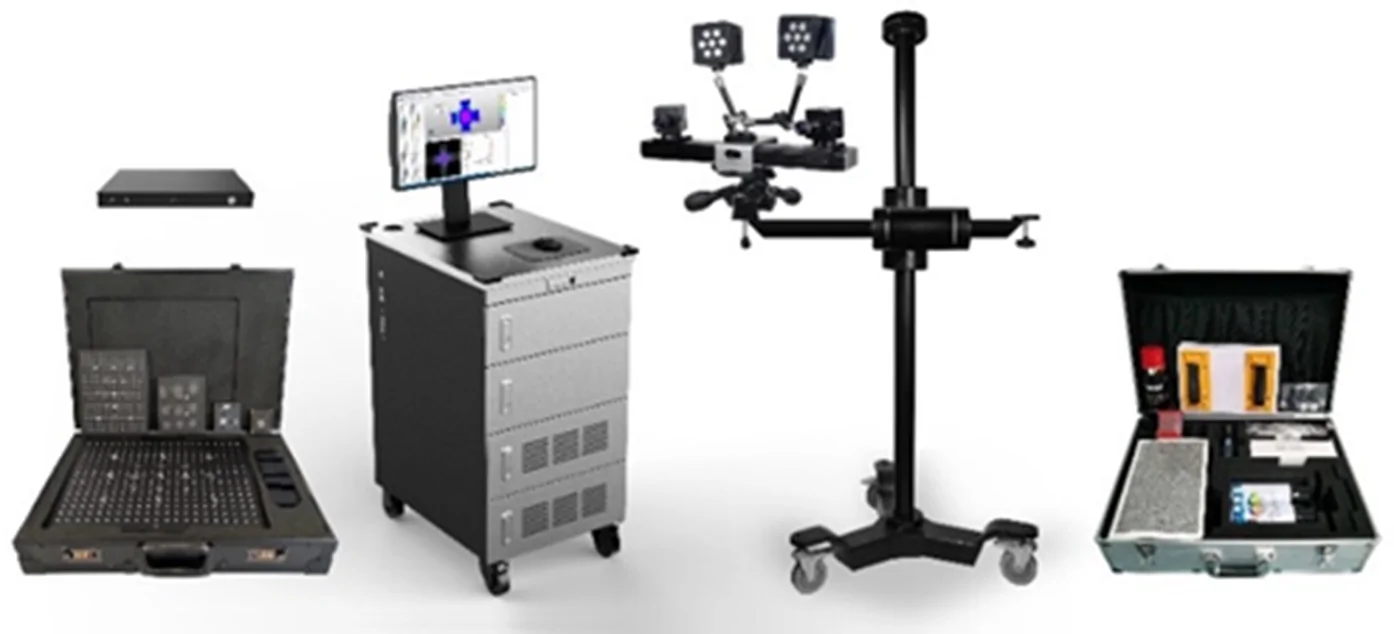
XTDIC measurement system.

### Results and analysis

In this study, the metal mesh and TSL surface support methods were comparatively evaluated based on peak load-bearing capacity, displacement response, local strain evolution, and failure characteristics. The experimental results are shown in Fig 4.

**Fig 4:**
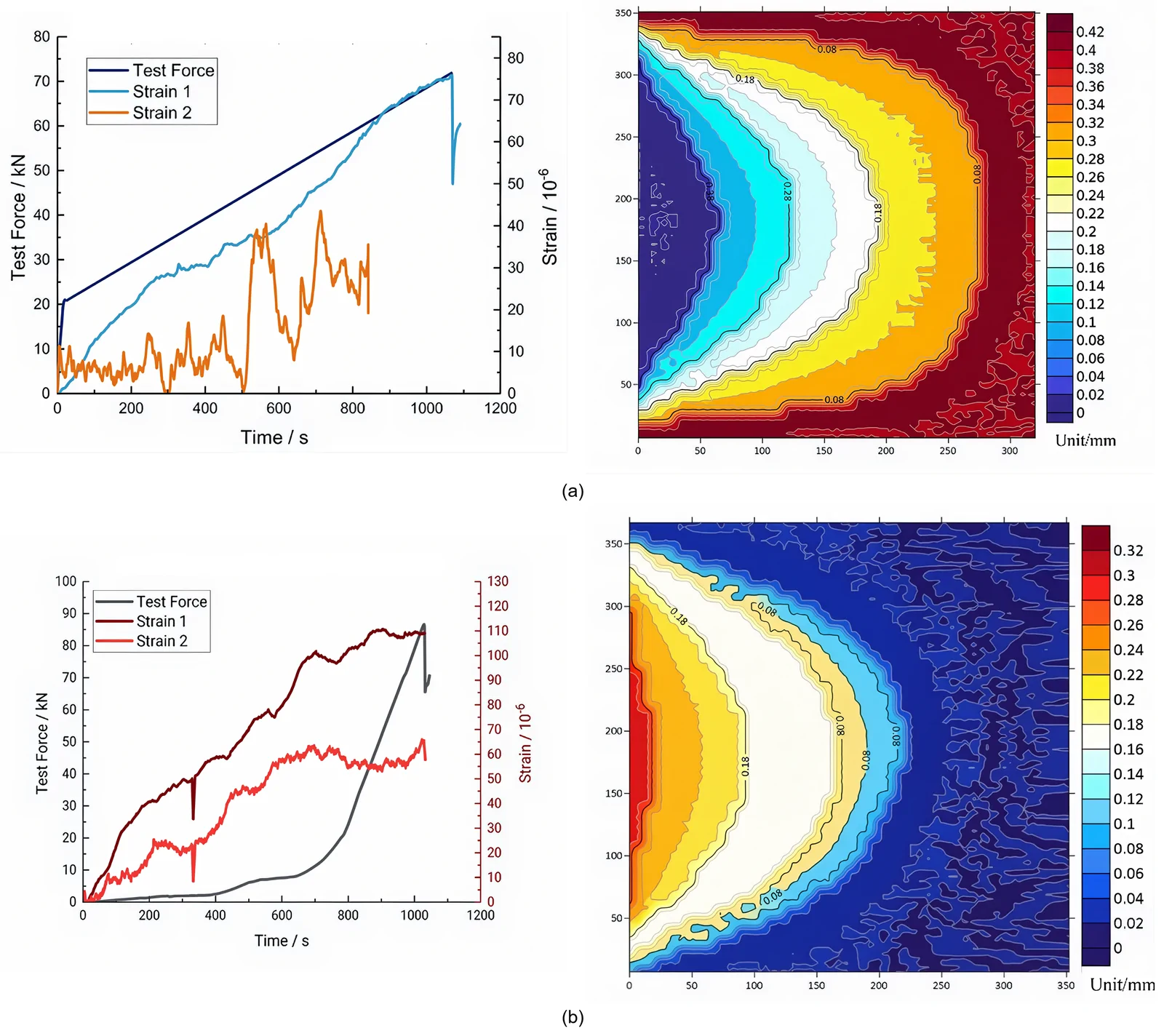
Force–strain–time responses and surface displacement contours of coal pillars with metal mesh and TSL support. (a) Metal mesh. (b) TSL.

As shown in Fig 4, the specimen supported by metal mesh reached a peak load of approximately 70 kN. During the early loading stage, the test force generally increased with time. However, the strain responses at the two measuring points showed clear differences. One strain curve exhibited multiple sudden increases and decreases between 500 and 850 s, while the other continued to increase during the later loading stage and rapidly decreased when approaching the peak load. The high-displacement regions were mainly concentrated in the middle areas of the roadway roof and floor and at the intersections between the roof/floor and sidewalls. The relatively dense contour lines indicate that localized deformation developed earlier in these regions. The metal mesh mainly provides surface control through tensile resistance, bending deformation, and containment of loose rock blocks. Before significant loosening or detachment of the rock mass occurs, its ability to restrain continuous surface deformation is relatively limited.

Under TSL support conditions, the specimen reached a peak load of approximately 85 kN, which was about 21.4% higher than that of the metal mesh group. The strain responses at both measuring points showed an overall continuous increase, with the final strain values reaching approximately $1.10\times 10^{-4}$ and $6.0\times 10^{-5}$, respectively. The maximum surface displacement of the specimen was about 0.32 mm, representing a reduction of approximately 23.8% compared with the 0.42 mm observed for the metal mesh group. The high-displacement regions were still mainly located in the middle areas of the roof and floor and near the sidewall corners, indicating that TSL did not change the primary load-bearing locations of the specimen. However, the continuous spray-on layer may reduce localized displacement concentration through surface bonding and membrane stress transfer, resulting in more continuous and coordinated deformation of the shallow coal–rock surface.

As shown in Fig 5, the metal mesh-supported specimen mainly exhibited longitudinal splitting of the coal pillar and local crushing at the ends during vertical loading. With increasing load, the coal pillar underwent lateral expansion under compression. Under lateral confinement, tensile stresses gradually developed inside the coal pillar, leading to longitudinal cracks extending along the loading direction. Meanwhile, stress concentration occurred at the contact zones between the coal pillar and the roof/floor, resulting in local crushing and particle spalling near the corners. After failure of the coal pillar, the loosened blocks were restrained by the metal mesh. The mesh underwent local tensile deformation and bending as the coal pillar deformed, thereby limiting further detachment of fragmented coal into the roadway space. These observations indicate that, in this test, the primary role of metal mesh was post-failure containment and block restraint, while its ability to limit early crack propagation and continuous lateral deformation of the coal pillar was relatively limited.

**Fig 5:**
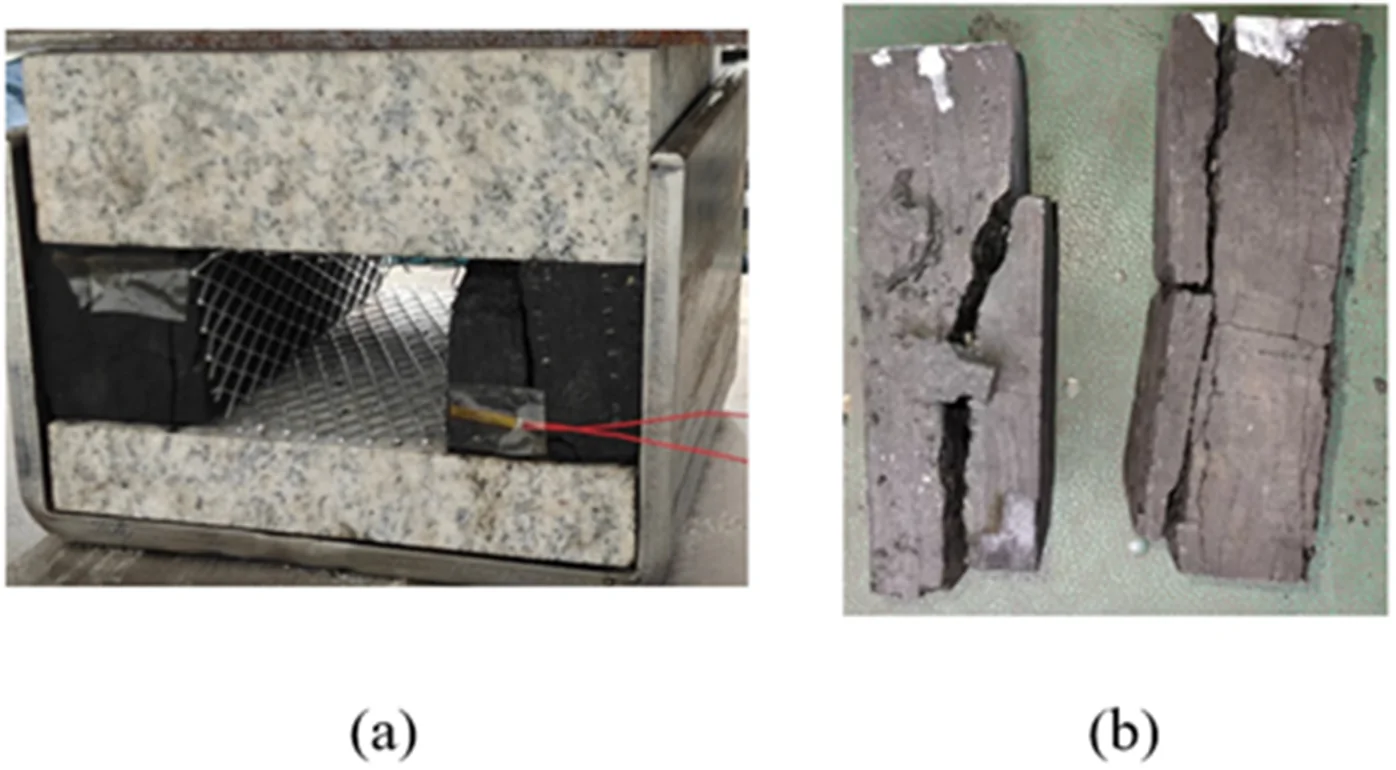
Failure characteristics of the metal mesh-supported specimen. (a) Deformed coal pillar with metal mesh support. (b) Fragmentation of the coal pillar in the metal mesh-supported specimen.

As shown in Fig 6, the TSL-supported specimen developed multiple longitudinal microcracks as the load increased. Crushing at the corners and particle detachment occurred near the contact zones between the coal pillar and the roof/floor due to stress concentration. With further lateral expansion of the coal pillar, irregular tensile cracks appeared in the spray-on layer. After local failure of the spray-on layer, the split and fragmented coal blocks were pushed toward the roadway void, but no overall collapse occurred.

**Fig 6:**
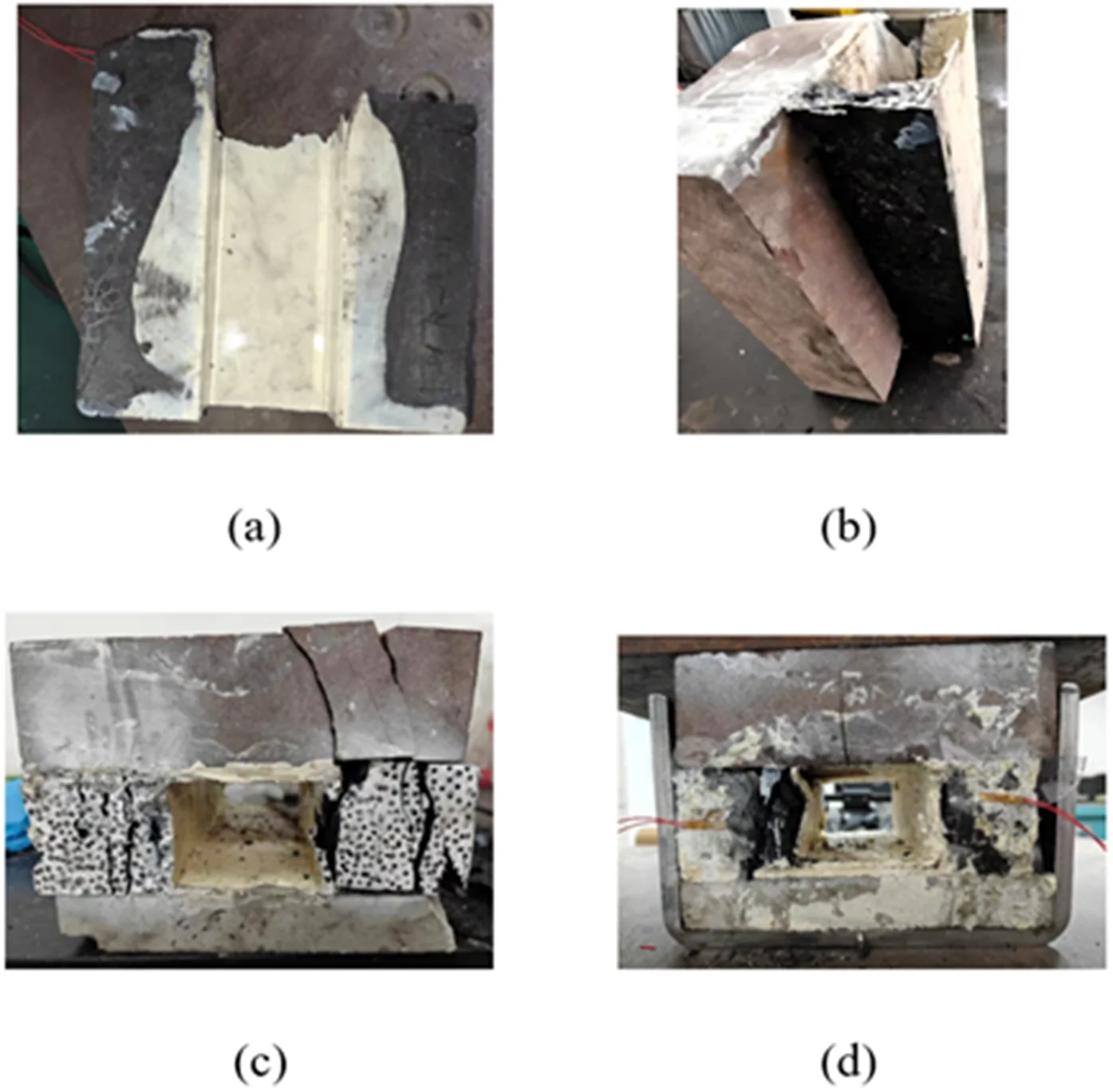
Failure characteristics of the TSL-supported specimen. (a) Failure of the TSL layer on the roof after testing. (b) Appearance of the TSL layer after testing. (c) Raw material before TSL application. (d) TSL application on the specimen surface.

From the perspective of rock mechanics, the support mechanism of TSL can be generally described in three stages: “initial bonding reinforcement–intermediate flexural restraint–late-stage thin-shell/load-carrying membrane interaction.” After curing, the spray-on layer bonds with the surrounding rock surface and may penetrate into shallow cracks, forming a relatively continuous confinement system for loose rock blocks. As surrounding rock deformation increases, the spray-on layer and the bonded shallow rock mass deform together. Through combined tensile, bending, and shear actions, they help restrain crack opening and lateral expansion. During the stage of larger deformation, even when local debonding or cracking occurs, the remaining continuous spray-on layer and the constrained shallow rock mass may still exhibit certain thin-shell or load-carrying membrane behavior. Compared with the discrete containment mechanism of metal mesh, the main feature of TSL is that its continuous coverage and interfacial bonding allow it to participate earlier in controlling deformation of the shallow surrounding rock.

## Numerical analysis of TSL support behavior in mining roadways

### Engineering-scale numerical model

Based on the geological conditions of a roadway at a mining face of Hongliulin Mining Company, Shaanxi Coal Group, a three-dimensional numerical model was established using FLAC3D 6.0. The main objective of the model was to compare the differences in surrounding rock displacement and stress responses between metal mesh and TSL surface support under identical bolt–cable primary support conditions. The surrounding rock was simulated using the Mohr–Coulomb ideal elastic–plastic model. The physical and mechanical parameters of each rock layer were determined according to geological data from the mining area and laboratory rock mechanical test results. It should be noted that the purpose of the numerical analysis was not to develop a rigorously validated predictive model, but rather to further investigate the influence trends of different surface support methods on surrounding rock responses at the engineering scale based on the support mechanism differences identified from the physical tests.

The TSL thickness was set as 5 mm. Since the thickness of the polymer thin spray-on layer is at the millimeter scale and much smaller than the roadway dimensions, its primary mechanical effects are considered to involve in-plane tensile resistance, local bending, and interfacial constraint between the spray-on layer and surrounding rock. Therefore, shell structural elements in FLAC3D were adopted to represent the TSL in an equivalent manner. This treatment does not consider TSL as equivalent to thick shotcrete or a rigid lining; rather, it is intended to describe the surface constraint and local bending response induced by continuous thin-layer coverage. In the numerical model, the TSL thickness was set to 5 mm, with an elastic modulus of 600 MPa, Poisson’s ratio of 0.25, tensile strength of 14 MPa, and an equivalent interface bonding strength of 1.63 MPa between the TSL and surrounding rock. The above parameters were adopted from the parameter system of a previous engineering-scale equivalent model. They were used to evaluate the influence of continuous thin-layer confinement on roadway surrounding rock responses and should not be regarded as directly transferable material design parameters.

The engineering-scale model had dimensions of 100 m (x-direction) × 50 m (y-direction) × 50 m (z-direction). The x-direction was set along the roadway axis. The roadway had a width of 5.0 m and a height of 3.5 m, and the buried depth was assumed to be 575 m, corresponding to the average value within the range of 550–600 m. The model was discretized using hexahedral elements. Since the deformation and stress redistribution of the surrounding rock after roadway excavation are mainly concentrated near the roadway boundary, the mesh was refined according to the principle of gradually increasing element size with increasing distance from the roadway. The element size in the near-wall region was approximately 0.5 m and gradually increased to about 2.0 m in the far field. This mesh arrangement improves the spatial resolution of the calculated responses around the sidewalls, roof, and floor while reducing unnecessary elements in regions far from the roadway. Sufficient distance was maintained between the model boundaries and the roadway to minimize the influence of boundary constraints on the near-field roadway responses. The main physical and mechanical parameters of the rock strata are listed in Table 5.

**Table 5:** Physical and mechanical parameters of the main rock strata.

Rock stratum	Thickness (m)	Density ($kg\cdot m^{-3}$)	Bulk modulus (GPa)	Shear modulus (GPa)	Tensile strength (MPa)	Poisson’s ratio	Internal friction angle (°)
Main roof (sandstone)	6	2650	16.3	12.5	4.3	0.30	38
Coal seam (No. 4−2 coal)	3.5	1400	1.5	0.8	0.4	0.25	30
Immediate floor (sandy mudstone)	0.5	2550	12.1	9.5	2.6	0.26	37
Main floor (carbonaceous mudstone)	1.5	2550	4.0	2.5	0.5	0.25	28

To ensure comparability between the two surface support conditions, the engineering-scale model adopted identical bolt–cable primary support parameters. The cable bolts had a diameter of 22 mm and a length of 8300 mm, with a spacing of 1150 mm × 900 mm. The rock bolts had a diameter of 22 mm and a length of 2400 mm, with a spacing of 830 mm × 900 mm. The bolts and cable bolts were simulated using cable structural elements in FLAC3D, and full-length anchorage was represented by assigning grouting bonding parameters along the entire bolt length. The two comparison cases used exactly the same bolt–cable arrangement, surrounding rock parameters, roadway geometry, initial stress conditions, and boundary conditions, thereby minimizing the influence of differences in the primary support system on the evaluation of surface support performance. The model was discretized using hexahedral elements. Local mesh refinement was applied in the vicinity of the roadway boundary, with an element size of approximately 0.5 m, gradually transitioning to approximately 2.0 m in the far field. Based on this model, two comparison cases were established: Case A with metal mesh surface support and Case B with TSL surface support. Except for the surface support type, all other conditions, including roadway geometry, surrounding rock parameters, boundary conditions, and anchorage parameters, were kept identical. Therefore, the differences between the two cases were considered to represent the overall influence of changing the surface support method under the same primary support conditions (Fig 7 and 8).

**Fig 7:**
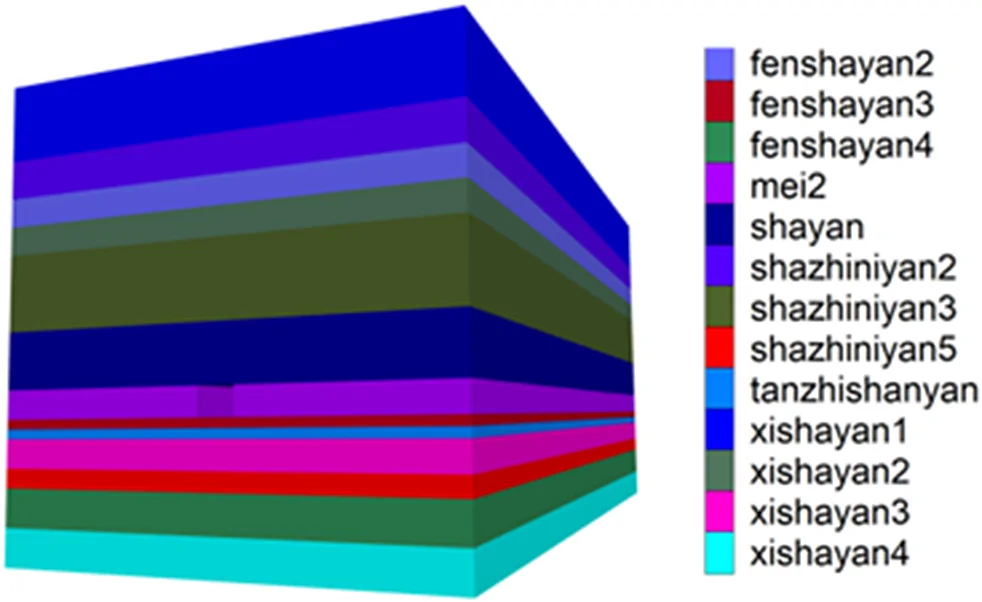
Model stratification.

**Fig 8:**
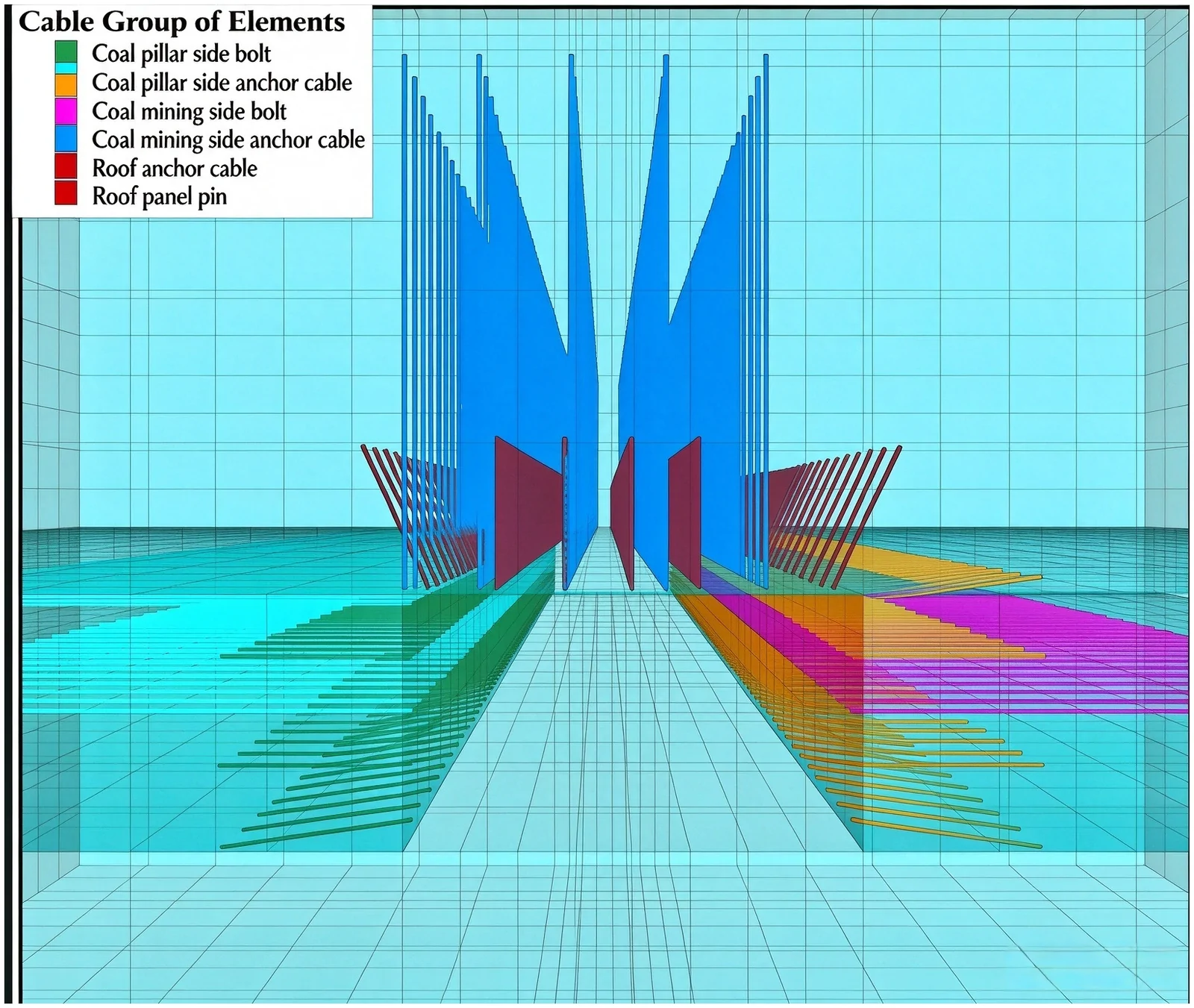
Roadway support.

The boundary conditions were set as follows. Normal displacement constraints were applied to the four lateral boundaries of the model (*x* = 0, *x* = 100 m, *y* = 0, and *y* = 50 m) to restrict horizontal movements. The bottom boundary (*z* = 0) was fully constrained, with displacement fixed in all directions. In the model coordinate system, the x-direction represents the roadway axis, the y-direction represents the horizontal transverse direction, and the z-direction represents the vertical direction. A uniformly distributed load of 14.38 MPa was applied on the top boundary to represent the self-weight of the overlying strata, corresponding to the vertical in-situ stress ($\sigma_{z}=14.38$ MPa). According to the in-situ stress data of the mining area, the maximum horizontal principal stress was approximately parallel to the roadway axis. A lateral pressure coefficient of 1.1 was adopted, resulting in an axial horizontal stress of $\sigma_{x}=15.82$ MPa. The transverse horizontal stress was assigned based on the measured in-situ stress conditions of the mining area. After establishing the three-dimensional initial stress field, an initial equilibrium calculation was first performed before roadway excavation.

### Comparison of surrounding rock responses with metal mesh and TSL support

In this section, with identical bolt–cable primary support parameters, the differences between Case A (metal mesh surface support) and Case B (TSL surface support) are compared in terms of sidewall horizontal displacement, roof and floor vertical displacement, and stress distribution.

Fig 9 shows that the roadway sidewalls exhibited similar anti-symmetric displacement characteristics under both support conditions. The maximum horizontal displacement was approximately 148 mm for the metal mesh support case and about 97 mm for the TSL support case, representing a reduction of approximately 34.1% in the displacement peak. These results indicate that TSL mainly reduces the magnitude of sidewall convergence and decreases the extent of the high-displacement zone near the roadway surface, while it does not change the basic deformation pattern of inward sidewall convergence.

**Fig 9:**
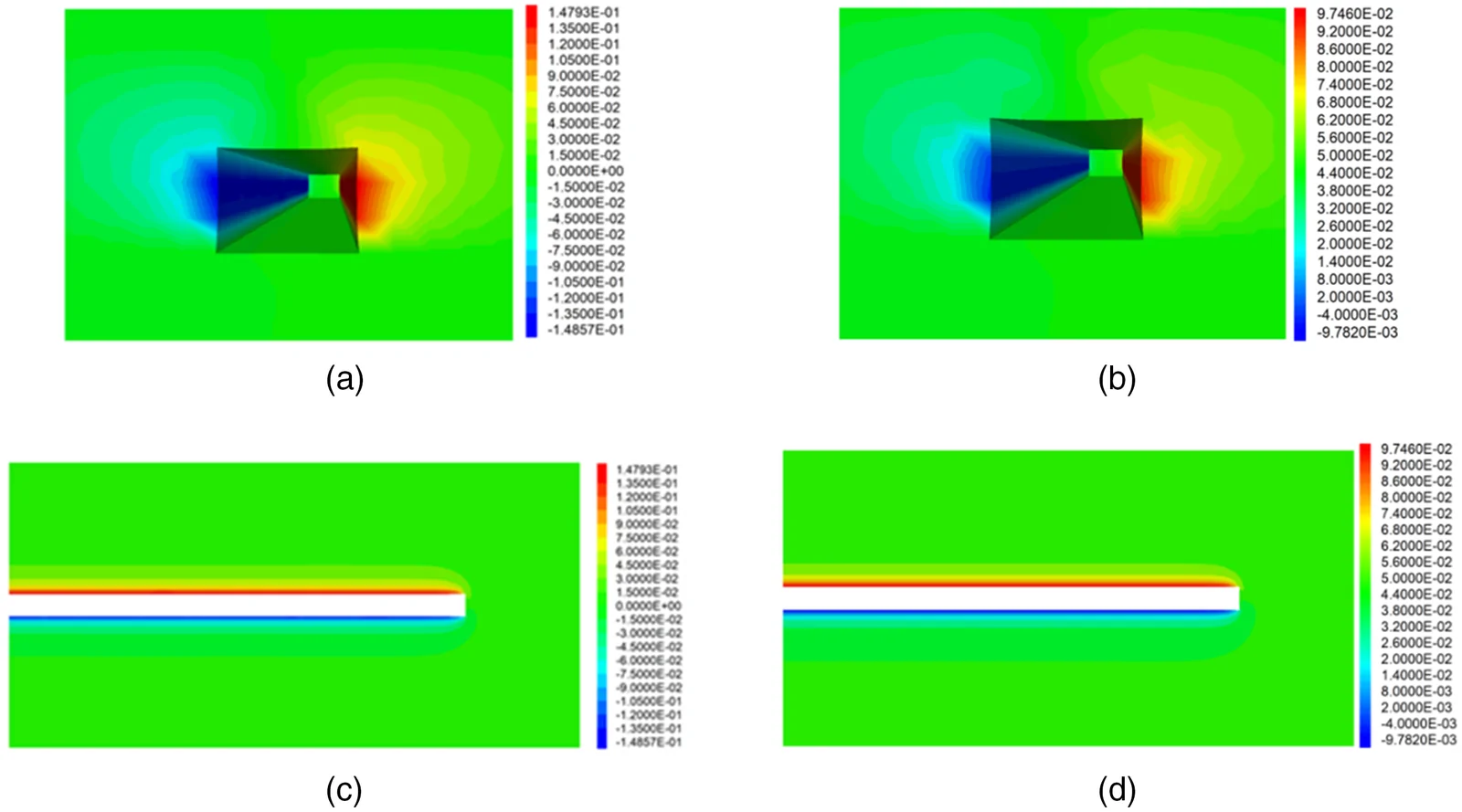
Horizontal displacement contours of the roadway sidewalls. (a) Horizontal displacement contour of the roadway with metal mesh support (yz plane). (b) Horizontal displacement contour of the roadway with TSL support (yz plane). (c) Horizontal displacement contour of the roadway with metal mesh support (xz plane). (d) Horizontal displacement contour of the roadway with TSL support (xz plane).

As shown in Fig 10, replacing metal mesh surface support with TSL support resulted in a reduction of the high-displacement zones in both the roof and floor. The peak floor heave decreased by approximately 6.3%, while the peak roof subsidence decreased by approximately 11.0%. Under the model conditions considered in this study, TSL provided a greater reduction in shallow roof deformation than in floor heave (Fig 11).

**Fig 10:**
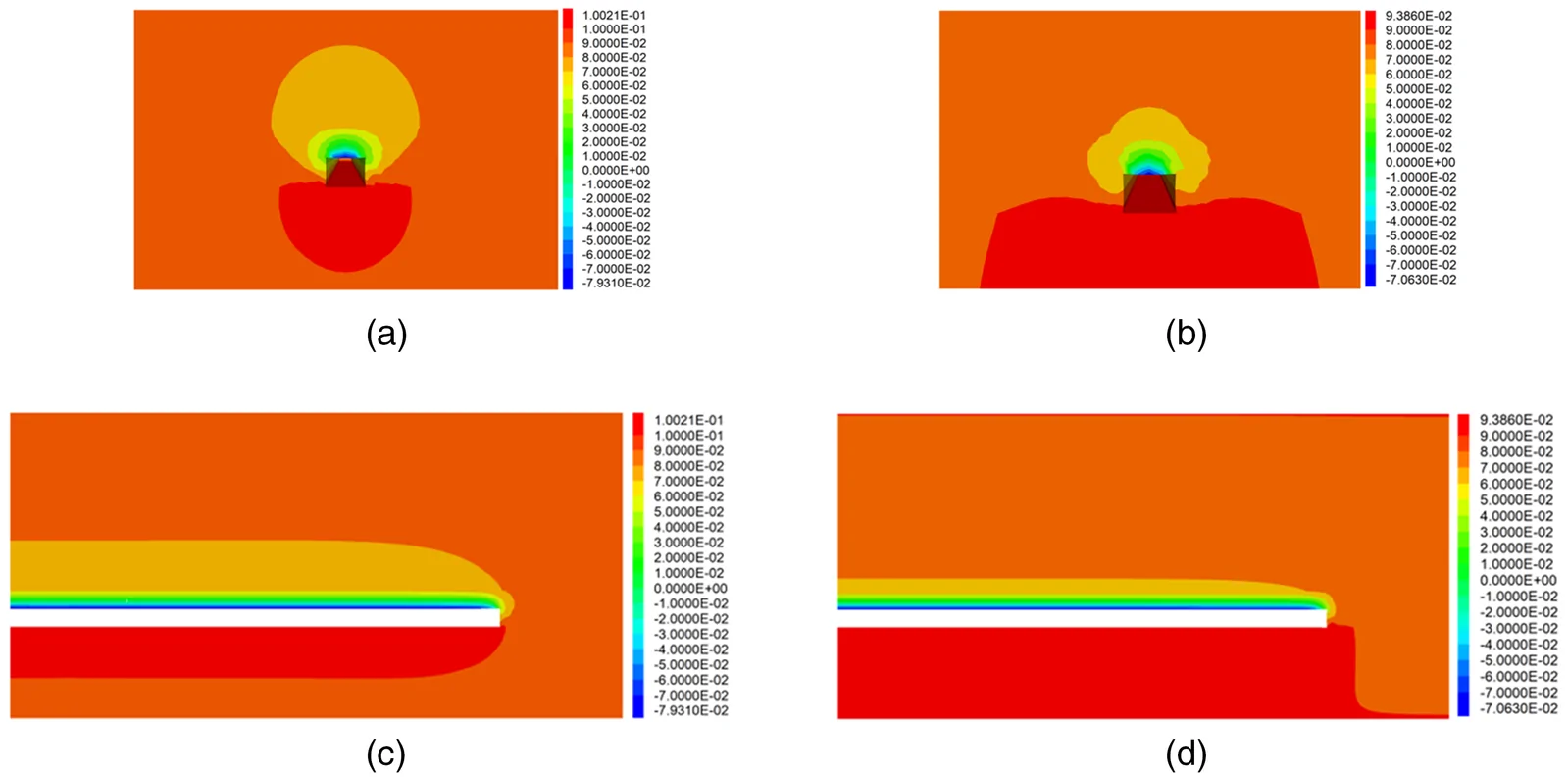
Vertical displacement contours of the roadway with metal mesh and TSL support. (a) Vertical displacement contour of the roadway with metal mesh support (yz plane). (b) Vertical displacement contour of the roadway with TSL support (yz plane). (c) Vertical displacement contour of the roadway with metal mesh support (xz plane). (d) Vertical displacement contour of the roadway with TSL support (xz plane).

**Fig 11:**
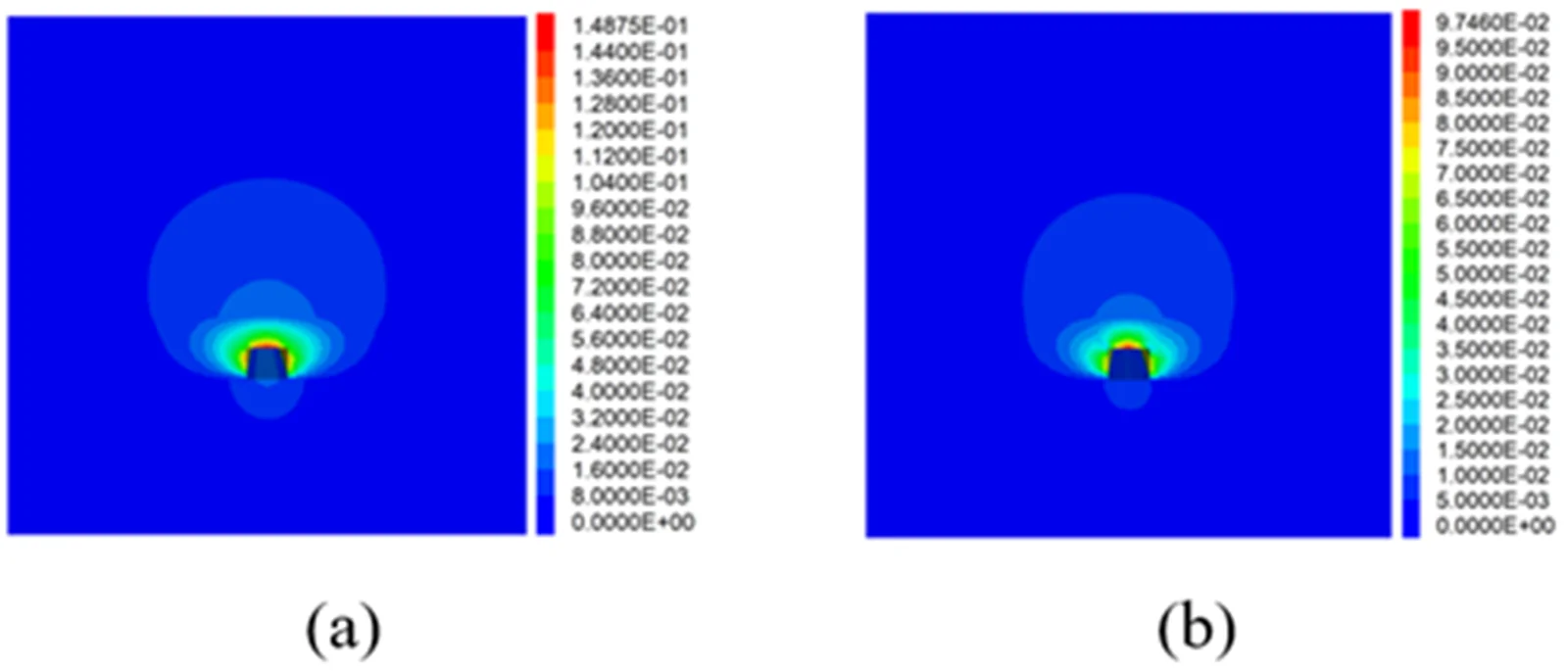
Total displacement contours of the roadway with metal mesh and TSL support. (a) Total displacement contour of the roadway with metal mesh support (yz plane). (b) Total displacement contour of the roadway with TSL support (yz plane).

Fig 12 presents the distribution characteristics of vertical stress in the roadway under metal mesh and TSL surface support conditions. In both cases, the areas near the sidewalls exhibited relatively high vertical stress concentrations. After replacing metal mesh surface support with TSL support, the extent of the high-stress zone was relatively reduced, the contour transitions became smoother, and the tendency of high-stress zones to extend deeper into the surrounding rock and along the roadway axis was weakened.

**Fig 12:**
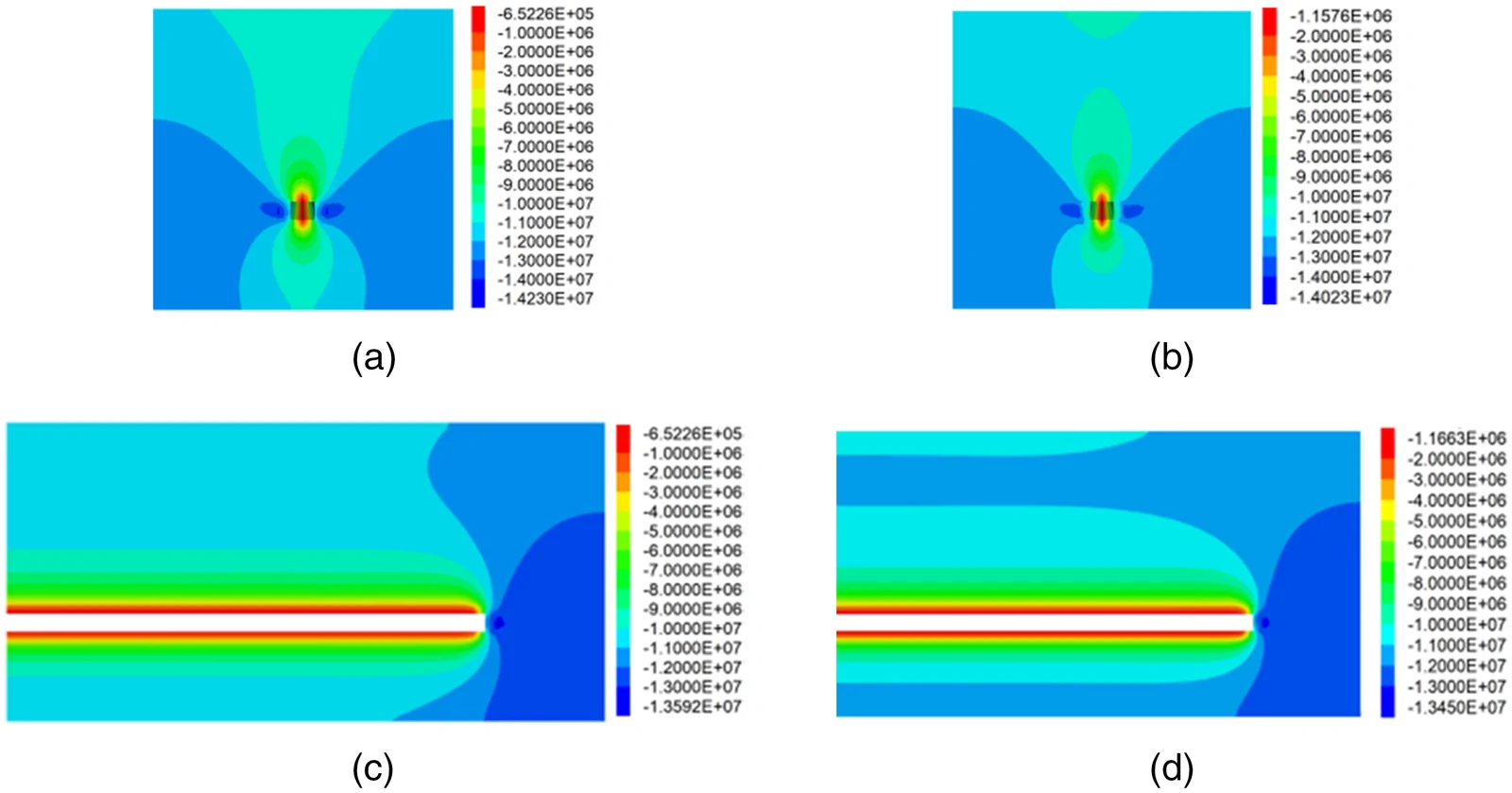
Vertical stress contours of the roadway with metal mesh and TSL support. (a) Vertical stress contour of the roadway with metal mesh support (yz plane). (b) Vertical stress contour of the roadway with TSL support (yz plane). (c) Vertical stress contour of the roadway with metal mesh support (xz plane). (d) Vertical stress contour of the roadway with TSL support (xz plane).

Fig 13 illustrates the distribution characteristics of horizontal stress at the sidewalls under the two support conditions. After replacing metal mesh surface support with TSL support, the high horizontal stress zones near the sidewalls became more concentrated, and the stress transition from the near-wall region to the deeper surrounding rock was relatively smoother. The results on the xy plane also show that the high-stress zones were mainly distributed closer to the roadway boundary, with a reduced tendency to extend into the deeper surrounding rock.

**Fig 13:**
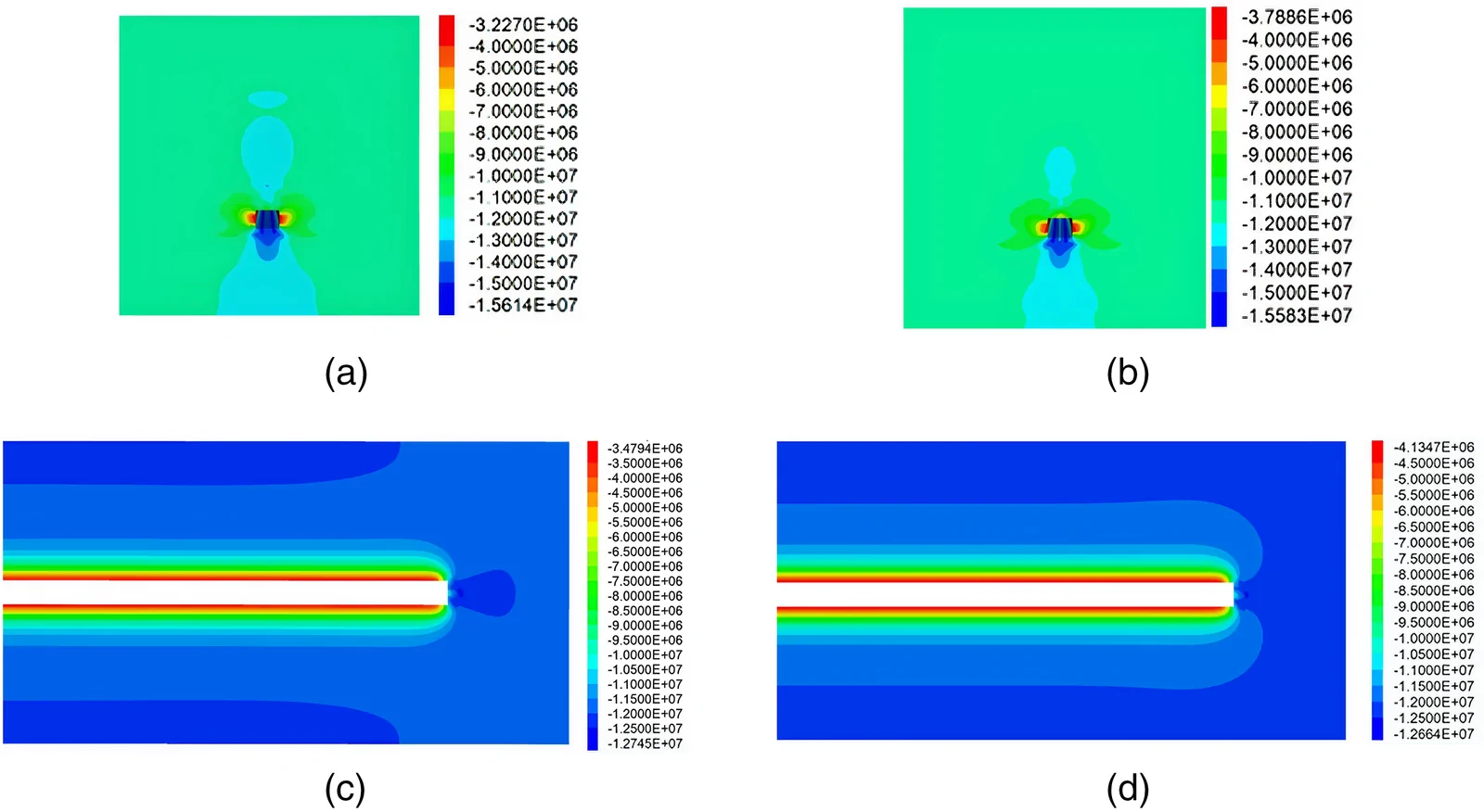
Horizontal stress contours of the roadway with metal mesh and TSL support. (a) Horizontal stress contour of the roadway with metal mesh surface support (yz plane). (b) Horizontal stress contour of the roadway with TSL support (yz plane). (c) Horizontal stress contour of the roadway with metal mesh surface support (xy plane). (d) Horizontal stress contour of the roadway with TSL support (xy plane).

In summary, the stress redistribution patterns after roadway excavation were generally consistent under the two support conditions, with stress release occurring near the roadway boundary and stress concentration developing in the regions adjacent to the sidewalls. After replacing metal mesh surface support with TSL support, the high-stress zones showed a relatively reduced extent and smoother contour transitions in the present model. This suggests that TSL may enhance the load-sharing behavior of shallow surrounding rock by constraining near-wall lateral deformation. It should be emphasized that this conclusion is based on numerical predictions obtained from a continuum model with equivalent shell elements for TSL representation, and it has not yet been validated through long-term field monitoring.

### Implications for engineering application

The combined experimental and numerical results indicate that the two surface support methods exhibit different support characteristics. In the simulated roadway tests, the metal mesh-supported specimens achieved a peak load of approximately 70 kN and a maximum surface displacement of about 0.42 mm, whereas the TSL-supported specimens reached a peak load of approximately 85 kN and a maximum surface displacement of about 0.32 mm. These results suggest that, under the boundary and loading conditions considered in this study, continuous thin spray-on surface support showed improved load-bearing and deformation-control responses under the investigated conditions and shallow surface deformation control. The engineering-scale numerical model further showed that, under the same bolt–cable primary support conditions, the peak sidewall convergence, roof subsidence, and floor heave in the TSL-supported case were reduced by approximately 34.1%, 11.0%, and 6.3%, respectively, compared with the metal mesh-supported case. Both experimental and numerical results suggest that the continuous coverage and interfacial confinement provided by TSL may contribute to controlling shallow surrounding rock deformation. However, these findings represent relative comparisons under the specific test and model conditions considered in this study (Table 6).

**Table 6:** Main results and implications at different research stages.

Research stage	Key results	Conclusions
Simulated roadway tests	The peak loads of the metal mesh and TSL groups were approximately 70 and 85 kN, respectively; the maximum surface displacements were approximately 0.42 and 0.32 mm, respectively.	Under the test conditions considered, TSL exhibited relatively continuous surface confinement and deformation control effects.
Engineering-scale numerical analysis	Compared with metal mesh surface support, the peak displacements of the sidewalls, roof, and floor were reduced by 34.1%, 11.0%, and 6.3%, respectively.	Under the model conditions considered, TSL improved the response of the near-wall surrounding rock.

A field spray-on application test was conducted underground (Fig 14). The available photographic records confirm that the spraying operation was performed under actual roadway conditions. Combined with the field records, the test demonstrated that the material could be sprayed onto the roadway surface and form a continuous coverage layer, providing preliminary evidence of the feasibility of the construction process. According to the current mine management requirements, metal mesh and other safety components still need to be retained in specific areas or at certain heights. It should be emphasized that this field work was a process feasibility test rather than a parallel comparison with conventional metal mesh support. Since long-term mine pressure monitoring data under comparable conditions are not yet available, the test results cannot be regarded as confirmation that TSL has achieved full engineering validation of support performance.

**Fig 14:**
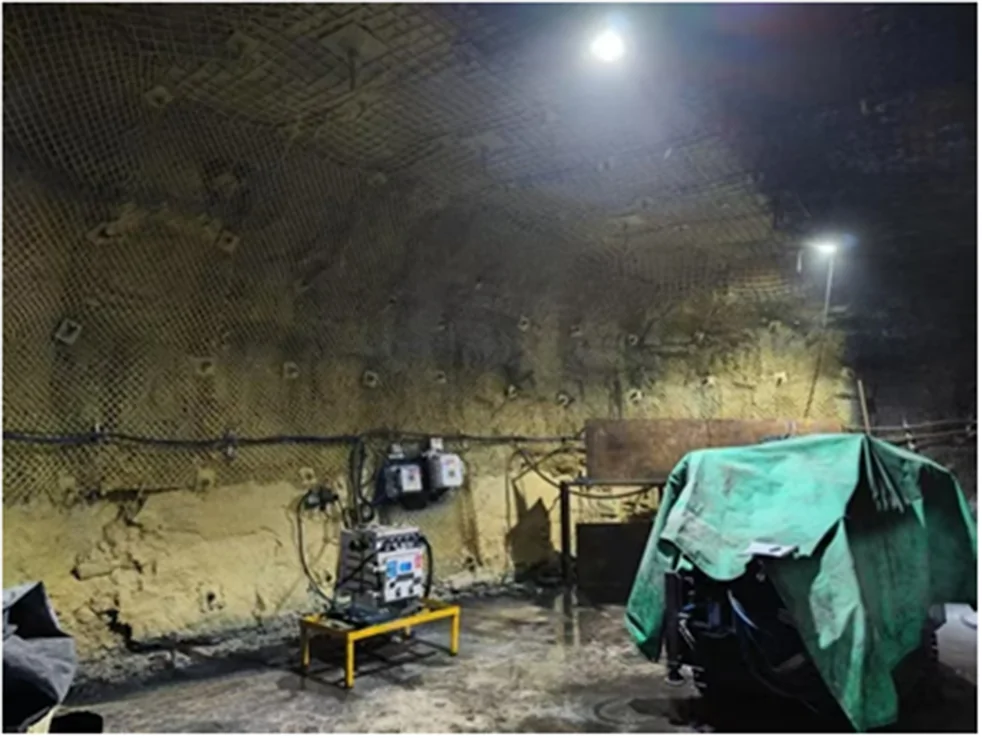
Underground field trial of TSL spraying with metal mesh retained in local areas.

From the perspective of safety, polymer-based TSL requires further specific evaluations before application in underground coal mines. These evaluations should at least include flame retardancy and flammability under high-temperature conditions, smoke density and toxic gas emissions during combustion, compatibility with mine water and common chemical media, and the retention of mechanical properties after long-term aging. The available data in this study do not include test results related to flame retardancy, smoke toxicity, chemical compatibility, or long-term aging performance. Therefore, no conclusions are drawn regarding the safety performance of the material under fire conditions, chemical exposure, or long-term service environments. Engineering application of TSL should be conducted on the basis of compliance with requirements for mining material approval, flame-retardant and antistatic properties, and other relevant safety regulations. Before TSL is formally considered as a replacement for metal mesh, additional specific safety tests and long-term field monitoring are required.

From an economic perspective, the available data do not include the material prices, material consumption per unit advance length or support area, crew size, labor hours per unit advance length, equipment depreciation and maintenance costs, or life-cycle maintenance costs required for a comparable cost assessment. Therefore, statements such as “more economical” or “significantly reducing costs,” which are not supported by quantitative data, are not included in this study. No conclusions are drawn regarding the economic advantages of TSL compared with metal mesh. Future economic evaluations should be conducted under the same roadway conditions by systematically accounting for material costs, labor costs, equipment costs, construction time, and maintenance costs per unit support area or unit advance length. A life-cycle cost comparison should also be performed considering the service period of the roadway.

## Conclusions

(1) Under identical model geometry, boundary conditions, and loading conditions, the metal mesh-supported specimens exhibited a peak load of approximately 70 kN and a maximum surface displacement of about 0.42 mm. The TSL-supported specimens reached a peak load of approximately 85 kN, representing an increase of about 21.4% compared with the metal mesh group, while the maximum surface displacement decreased to about 0.32 mm, corresponding to a reduction of approximately 23.8%. These results indicate that, under the test conditions considered, continuous TSL surface support may improve the deformation compatibility and overall load-bearing response of the coal–rock and surface support composite system.
(2) The two surface support methods exhibit different support mechanisms. Metal mesh mainly restrains further detachment of loosened blocks through tensile resistance, bending deformation, and containment effects. In contrast, TSL may rely on continuous coverage, interfacial bonding, and membrane stress transfer to participate in shallow surface confinement at relatively small deformation stages. During progressive deformation, it may exhibit flexural and thin-shell/load-carrying membrane interaction characteristics.
(3) Engineering-scale numerical analysis shows that, with identical roadway geometry, surrounding rock parameters, and bolt–cable support conditions, replacing metal mesh surface support with TSL support reduced the peak sidewall horizontal displacement, roof subsidence, and floor heave by approximately 34.1%, 11.0%, and 6.3%, respectively. The extent of the high-stress zones was also relatively reduced. These results represent the combined influence of changing the surface support method under the model conditions considered and require further validation through long-term field mine pressure monitoring.
(4) This study has several limitations. The small-scale tests were mechanism comparison experiments based on geometric similarity rather than a strictly complete similarity model, and the strength, stiffness, and time similarity requirements were not fully satisfied. The engineering-scale numerical model adopted a continuum approach with an equivalent thin-layer representation and could not explicitly simulate complex debonding and through-thickness tearing of the spray-on layer. In addition, specific data regarding economic performance, flame retardancy, smoke toxicity, chemical compatibility, and long-term aging behavior remain unavailable. Therefore, the conclusions of this study should be understood as relative comparisons between metal mesh and TSL surface support under the specific experimental and numerical conditions investigated.
